# Interval type-2 intelligent fuzzy vehicle speed controller design using headlamp reflection detection and an adaptive neuro–fuzzy inference system

**DOI:** 10.1371/journal.pone.0323913

**Published:** 2025-06-02

**Authors:** Seung-Min Ryu, Kang-Hyeon Choi, Hyuk-Jun Chang

**Affiliations:** School of Electrical Engineering, Kookmin University, Seoul, South Korea; Industrial University of Ho Chi Minh City, VIET NAM

## Abstract

In this study, we present an algorithm to estimate the distance between a vehicle and a target object using light from headlights captured by a camera. In situations with limited distance data, we also design a fuzzy controller using the adaptive neuro–fuzzy inference system (ANFIS). To enhance robustness against disturbances, the interval type-2 approach is used. For the distance estimation algorithm, the vehicle is positioned at predefined intervals from the target object, capturing images of the headlights at each point. The region of interest containing the light is extracted from each image and segmented by light intensity. Weighted values are then assigned to each segment based on intensity, producing an image value that correlates with the distance. This image-derived value is then used as distance data for the design of the fuzzy controller. The controller is implemented using the interval type-2 fuzzy logic toolbox in MATLAB/SIMULINK, with vehicle speed and image intensity values as inputs and control torque as the output to adjust vehicle speed. The noise from the vehicle speed sensor is treated as a disturbance, and the performance of the interval type-2 fuzzy controller is evaluated under these disturbance conditions. Additionally, fuzzy controllers are designed for vehicle positions between 41–43 m and 47–49 m, and these controllers are trained using ANFIS to function effectively across the entire 41–49 m range. Simulation results demonstrate that, with the controller integrated into the vehicle system, the vehicle is successfully controlled to reach the target position.

## Introduction

Headlights are vital components of vehicles, serving multiple critical functions. Under low-light situations, such as nighttime, headlights enhance driving stability by ensuring adequate visibility. During the day, they can serve as communication tools, signaling traffic conditions to vehicles ahead by blinking. The uses of headlights are abundant, and numerous studies are currently being performed across various fields, including autonomous driving systems, to improve convenience and safety.

High beams produce intense light that illuminates a wide area under dark road conditions. However, this intense light can create glare for oncoming drivers, potentially impairing their visibility. Intelligent headlights help prevent accidents using sensors to detect other vehicles and the surrounding environment and adjust the high beams accordingly [1–3]. Additionally, headlights are used for vehicle-to-vehicle communication. Through visible light communication, headlights can transmit various information, such as speed, location, and direction, to vehicles in front and behind [4–6]. Moreover, headlights are crucial for nighttime surveillance. While vehicles can be reliably detected by surveillance cameras during the day, identifying them becomes more difficult under dark conditions. Current research focuses on recognizing vehicles by detecting the light emitted from their headlights [7–9].

This study proposes an advanced control approach based on headlight detection. The vehicle’s position is estimated by detecting the light emitted from its headlights, and its speed is adjusted using a fuzzy controller. The goal is to implement the proposed method in future autonomous driving systems, such as adaptive cruise control (ACC). Autonomous vehicles typically use various sensors, including LiDAR and radar, to accurately measure vehicle position. As the technology for autonomous driving advances, both the data processing demands and costs associated with vehicles are expected to increase. This study addresses these challenges by proposing a solution that determines vehicle position using only a camera combined with a computational algorithm.

Vehicles generally travel at an average speed of 60 km/h on the roads, maintaining a safety distance of approximately 45 m. Although ACC systems are intended to maintain a constant distance from nearby vehicles, fluctuating traffic conditions typically make it difficult to maintain this spacing consistently. Conducting experiments under real road conditions poses significant challenges. To address these limitations, this study estimates vehicle positions by capturing headlamp reflections from a designated target at approximately 45 m and processing these images using MATLAB/SIMULINK.

Fuzzy theory mathematically captures the ambiguity inherent in human language [10]. Based on fuzzy theory, fuzzy control effectively addresses control problems suited to particular objectives [11, 12]. Fuzzy control also simplifies the management of complex mathematical models, enabling systems to achieve optimal performance based largely on expert experience [13, 14]. In this study, we focus on the simultaneous processing of light and vehicle speed, and the mathematical modeling is notably complex. Fuzzy control is used in this study because of its effectiveness in applications that do not require an exact model.

Neuro-fuzzy combines the learning capability of neural networks and the rule-based decision of fuzzy logic, enabling effective system control and its application across various fields [15, 16]. In [17], a neuro–fuzzy control method was used to manage a robotic exoskeleton driven by electromyogram signals from physically vulnerable individuals, including the elderly and disabled. While a quadrotor with four rotors provides enhanced stability, modeling and controlling are difficult. To address this control challenge, a previous study [18] introduced a fuzzy controller that used yaw, pitch, and roll values as inputs, with the power supplied to each rotor as outputs. Another study [19] shows that using a fuzzy controller in a DC–DC converter can improve performance compared to a traditional PID controller. Fuzzy controllers have also been applied in image processing. In [20], a method is proposed to enhance video quality through the use of a fuzzy controller to point cloud video. Additionally, a previous study [21] regulated vehicle speed using distance estimation obtained from headlamp reflection detection as input for the fuzzy controller. This study addresses the performance issue related to external disturbances, which is a limitation noted in another study [21], and enhances it by implementing ANFIS in situations where distance data are lacking.

In a previous study [21], a type-1 fuzzy controller is used, but it has limitations owing to its susceptibility to disturbances such as sensor noise. To mitigate this issue, this study employs an interval type-2 fuzzy controller, which is more robust against uncertainty [22–25]. In an interval type-2 fuzzy controller, the membership functions of input variables take values in a specified range. This characteristic enhances the controller’s ability to manage input uncertainty and improves the system’s performance. The study uses the interval type-2 fuzzy logic toolbox in MATLAB/SIMULINK [26, 27]. This vehicle’s dynamic equations are implemented in MATLAB/SIMULINK, where the interval type-2 fuzzy controller is used to regulate vehicle speed.

Previous studies have encountered challenges in obtaining vehicle distance data because the capture of light images is a necessary requirement. To overcome this issue, this study uses an ANFIS [28–30]. ANFIS integrates a Sugeno-based fuzzy inference system with an artificial neural network, allowing for the creation of new input–output models by learning from existing data. In this study, ANFIS is trained using input–output data from a fuzzy controller that takes vehicle speed and distance as inputs to produce control torque. This approach presents a method for designing an effective controller even when distance data are scarce.

This study explores vehicle position estimation through headlight light detection in “Vehicle position estimation using headlamp reflection detection” section and outlines the design of a vehicle dynamics model in “Vehicle dynamics model implementation” section. “Fuzzy controller design” section describes speed control using an interval type-2 fuzzy controller, while “Treatment of the limit of image data using an adaptive neuro–fuzzy inference system” section introduces a controller design method that uses ANFIS to address the challenge of insufficient distance image data. Lastly, “Discussion and conclusion” section presents the conclusions and offers suggestions for future research directions.

## Vehicle position estimation using headlamp reflection detection

In light illumination experiments, a KIA K7 is used as a vehicle. The headlights are adjusted to a low beam, and light reflections are captured using a camera. The low beams are mounted at a height of 719 mm from the ground, and the distance between the lights measuring 1454 mm. The maximum illumination distance is approximately 70 m, with a downward angle of 1.0%.

The vehicle is placed between 40 and 49 m from the target and moved in 1 m increments for each capture. The experiment is performed in an outdoor parking lot, where the light from the low beams is clearly visible on a building, and the light images are recorded using a camera located inside the vehicle.

**Fig 1:**
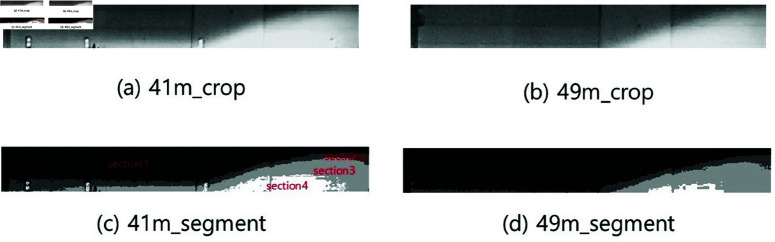
Region of interest and light segmentation in the image.

A camera mounted on the vehicle’s dashboard captures the image in black and white to facilitate image processing. Fig 1(a) and 1(b) show the regions of interest from the light. These areas are obtained by cropping the image from the top of the light’s cut-off line down to a specified size.

**Fig 2:**
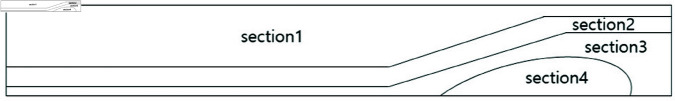
Detailed view of Fig 1(c).

Image segmentation is performed by normalizing the total size of the region of interest to 1, meaning the sum of all segmented regions equals 1. Fig 1(c) and 1(d) show the areas segmented by light intensity from Fig 1(a) and 1(b), respectively. The MATLAB/SIMULINK function imquantize() is used for segmentation, using threshold values of 140, 185, and 230. Non-light areas are excluded, and the image is divided into four sections based on these thresholds. The darkest area is designated as area 1, while the brightest is labeled as area 4, and so on. Further details of Fig 1(c) are shown in Fig 2.

The size of the brighter regions in images captured from closer distances is larger than those from further distances. For instance, area 1 at 41 m is larger than area 1 at 49 m, while area 4 at 41 m is smaller than area 4 at 49 m. To calculate image values based on distance, each segmented area is multiplied by weights of 1, 2, 3, and 4, respectively. Brighter sections are assigned higher weights, establishing a relationship in which the image value decreases as the distance increases. Table 1 presents the weighted values for each area along with their total sum. This total value represents the image value, which serves as an estimation for distance.

**Table 1:** Image values by distance.

Distance	41	43	45	47	49
area1	0.5739 × 1	0.6239 × 1	0.6469 × 1	0.6771 × 1	0.7101 × 1
area2	0.1359 × 2	0.1302 × 2	0.1256 × 2	0.1061 × 2	0.0940 × 2
area3	0.2055 × 3	0.1809 × 3	0.1810 × 3	0.1807 × 3	0.1697 × 3
area4	0.0848 × 4	0.0605 × 4	0.0446 × 4	0.0361 × 4	0.0261 × 4
total	1.8014	1.6870	1.6275	1.5758	1.5116

To convert the distance between vehicles into an image, first set the path of the image file. Then, load the images and adjust the distance value. Based on the distance value, select the appropriate image and return it. The pseudocode for selecting an appropriate image based on the input distance is as follows:

Function select image(distance)

image paths = [’path1’, ’path2’, ..., ’path9’]

For each path in image paths:

Read the image from the path

Store the image in the image list

distance = distance + 1

Switch(distance):

Case num:

Select images[num]

Return selected image

To output a specific value based on the brightness ratio of an image, the input image is preprocessed, and its histogram is calculated and normalized. The image is then segmented using threshold values and the pixel ratio for each group is calculated. Through this process, the final value is determined. The pseudocode for analyzing the input image and computing a weighted average score based on pixel value distribution is as follows:

Function multithresh(image)

img = Convert image to grayscale

(M, N) = Get the size of img

hist = Initialize an array of size 256 with zeros

For each pixel (i, j) in img:

hist[img(i, j) + 1] = hist[img(i, j) + 1] + 1

hist = hist / (M * N)

T = set threshold

seg I = Apply thresholding using T on img

total ratio 1-4 = Sum of hist

total score = total ratio1 * 1 + total ratio2 * 2 + total ratio3 * 3 + total ratio4 * 4

Return total score

## Vehicle dynamics model implementation

### Modeling

**Fig 3:**
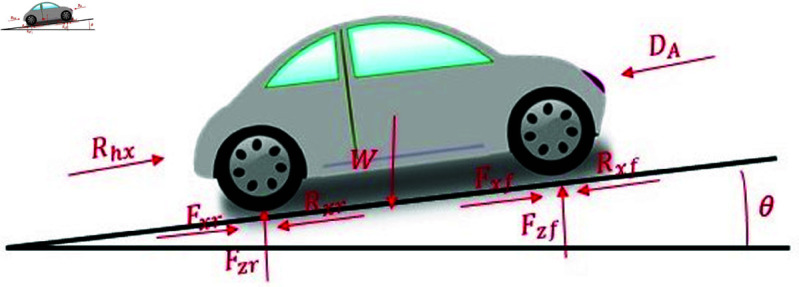
Longitudinal dynamics model of the vehicle.

We use the equations for longitudinal and rotational motion from [19] to develop and summarize a vehicle dynamics model. All motion equations are based on angular acceleration, as outlined in [19]. Eq (1) shows the vehicle’s acceleration divided by the wheel radius, reflecting angular acceleration while accounting for factors such as air resistance, gravity, traction, driving force, center of mass, rolling resistance, and other parameters. The longitudinal dynamic model of the vehicle, based on Eq (1), is depicted in Fig 3 [31].

Eq 2 is derived by applying Newton’s second law to the rotational motion and moment of the wheel. It illustrates the relationships between the wheel’s angular velocity, engine torque, brake torque, wheel radius, driving force, frictional torque, moment of inertia, and other factors. We assume both viscous and dry friction are present in the wheel.

$$\dot{\omega_{v}}=[-(0.5\rho C_{d}A)(r_{w}\omega_{v}+u_{w}{)}^{2}\;-fmcos\theta -mgsin\theta +N_{w}F_{z}\mu (\lambda )]/(mr_{w}),$$ (1)

$$\dot{\omega_{w}}=[-f_{w}F_{z}-b_{w}\omega_{w}-F_{z}r_{w}\mu (\lambda )+T]/J_{w}.$$ (2)

All parameters used in the model are presented in Table 2. Moreover, wheel slip λ is defined according to the vehicle’s acceleration and deceleration conditions as follows:

**Table 2:** Vehicle dynamics model parameters.

Parameter	Name	Value
*N*_*w*_	Number of drive wheel	2
f	Wheel friction coefficient	0.01
*C*_*d*_	Drag coefficient	0.28
*b*_*w*_	Wheel width	0.0*m*
*u*_*w*_	Wind velocity	0.0*m*/*s*
*r*_*w*_	Wheel radius	0.316*m*
θ	Slope angle	0.0
m	vehicle mass	1613*kg*
g	Gravitational acceleration	$9.81{\text{m}}^{2}$
*F*_*z*_	Vertical force on the wheel	3560*N*
p	Air density	$1.202kg/{\text{m}}^{2}$
A	Cross-sectional area	$1.95{\text{m}}^{2}$
*I*_*w*_	Wheel inertia	$0.65kg*{\text{m}}^{2}$
*I*_*e*_	Engine inertia	$0.429kg*{\text{m}}^{2}$
*r*_*g*_	Gear ratio	9.5285
*f*_*w*_	Wheel friction force	0.0*N*

$$\lambda =\frac{\omega_{w}-\omega_{v}}{\omega_{w}},\;\text{if}\;\omega_{w}>\omega_{v}\;(\text{accelerating}),$$ (3)

$$\lambda =\frac{\omega_{w}-\omega_{v}}{\omega_{v}},\;\text{if}\;\omega_{v}>\omega_{w}\;(\text{braking}).$$ (4)

The friction coefficient μ is a function of wheel slip λ, and the relationship is expressed as follows:

$$\mu (\lambda )=\sum_{i=0}^{n}c_{i}\lambda^{i}.$$ (5)

The coefficients *c*_*i*_ are obtained through curve fitting in Eq 5. The values for a 6th-degree polynomial are as follows:

$$[c_{0},c_{1},c_{2},c_{3},c_{4},c_{5},c_{6}]=[-68.593,238.216,\;-324.819,219.283,-75.58,12.088,-0.0068].$$ (6)

### Implementation

**Fig 4:**
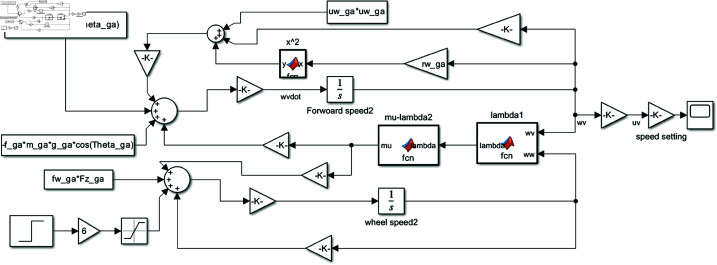
Vehicle dynamics model implemented in Simulink.

Fig 4 shows the vehicle dynamics model implemented in MATLAB/SIMULINK, based on Eqs 1 and 2. The calculations for Eqs 3 and 4 are performed in the lambda1 block, while the mu-lambda2 block handles Eq 5. The input torque has been adjusted to ensure a constant vehicle speed. The output angular velocity $\omega_{v}$ is multiplied by the wheel radius *r*_*w*_ to convert it into the vehicle’s speed.

**Fig 5:**
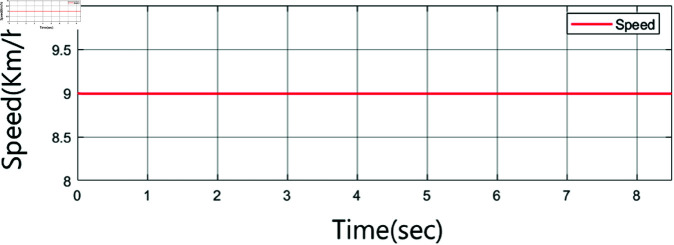
Output velocity of the vehicle dynamics model.

This study aims to control autonomous vehicles to match the speed of the vehicle in front. For instance, if the initial relative speed to the front vehicle is 9 km/h, this speed must be reduced to 0 km/h. Specifically, the vehicle’s speed will be decelerated from 9 to 0 km/h through braking in the simulation based on experimentation performed in this study. The initial speed output of the vehicle model is shown in Fig 5.

## Fuzzy controller design

### Interval type-2 fuzzy controller

In previous research, a type-1 fuzzy controller is used. Type-1 fuzzy logic has membership functions with fixed single values, which makes computation simple and real-time processing efficient. However, the performance of type-1 fuzzy degrades in highly uncertain environments. As a result, previous studies have the limitation of being vulnerable to disturbances such as sensor noise. To address this issue, this study employs an interval type-2 fuzzy controller, which exhibits superior robustness against uncertainty. Since type-2 fuzzy logic has membership functions with boundaries, it is more resistant to disturbances than type-1 fuzzy logic. Therefore, this study aims to design a controller that is robust to disturbances such as sensor noise using a type-2 fuzzy controller.

**Fig 6:**
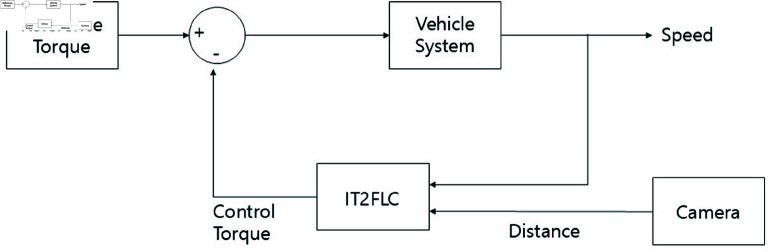
Overall system block diagram.

**Fig 7:**
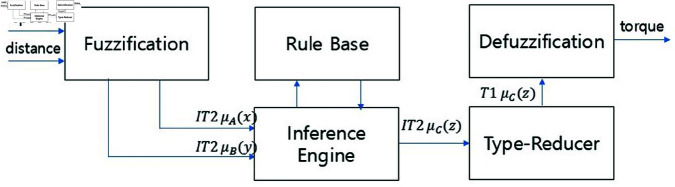
Interval type-2 fuzzy system block diagram.

The fuzzy controller is designed to use vehicle speed and distance data as inputs while controlling torque as its output. To address disturbances, such as noise that may arise during vehicle speed measurements from sensors, an interval type-2 fuzzy controller is used. The interval type-2 fuzzy controller has a low computational load, which makes it suitable for real-time processing. In addition, even if the number of rules increases, the computational complexity does not significantly increase, thereby allowing the interval type-2 fuzzy controller to operate within typical sampling times. Experiments are performed in MATLAB/SIMULINK, using the interval type-2 fuzzy logic toolbox. The fuzzy inference system (FIS) is based on the Sugeno approach, with the Karnik–Mendel (KM) algorithm used for type reduction and defuzzification. The overall system structure is shown in Fig 6, while the fuzzy control system is shown in Fig 7.

**Fig 8:**
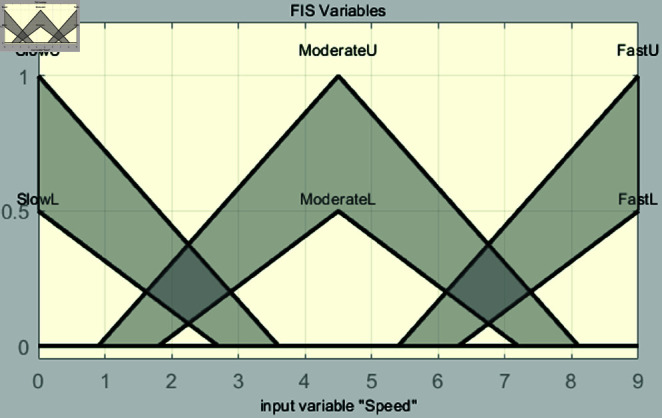
Interval type-2 membership function for speed.

**Fig 9:**
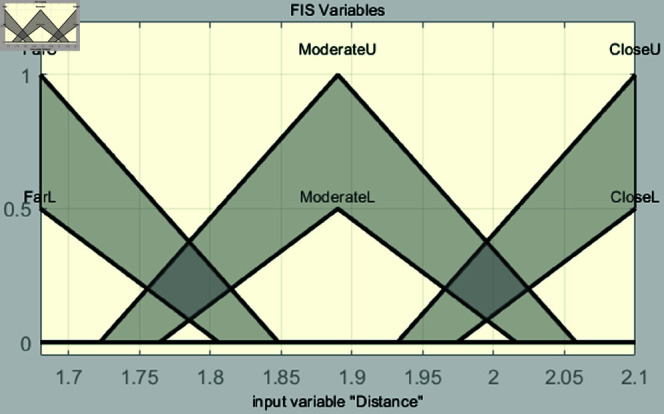
Interval type-2 membership function for distance.

In the fuzzification stage, the system establishes input membership functions for distance and speed, along with an output membership function for control torque. The input for distance is derived from image values in Table 1. Membership functions are defined as far, moderate, and close. For speed, the membership functions are categorized as slow, moderate, and fast, covering an input range from 0 to 9. This setup is used to perform vehicle speed control simulations based on experiments in the range of 40–49 m. Each membership function is shown in Figs 8 and 9.

The input variable *x*, representing the image value associated with distance, is used in Fig 8. When converting the distance to an image value, effective vehicle control can be achieved within the range of 1.68 to 2.1. Therefore, the range of the input variable *x* is set from 1.68 to 2.1, and this range is divided into six intervals to configure the parameters of the input variable. Triangular membership functions are used. The triangular membership function is mathematically simple and easy to implement, which is why it is commonly used in fuzzy system design. Its definition is intuitive, allowing for quick system modeling and tuning, and its calculations are simple and fast. This provides a significant advantages for real-time control systems. In addition, the triangular membership function effectively handles uncertainty and ambiguity while maintaining clear boundaries, particularly in the intermediate values. Since the objective of this study is to improve system performance by maintaining robust control performance in uncertain situations and enabling fast real-time calculations, the triangular membership function is selected. The triangular membership function is expressed as follows:

$$\mu_{A}(x)=\left\{ \begin{array}{cc} 0 & \text{if }x\leq p_{l}\text{ or }x\geq p_{r}\\ \frac{x-p_{l}}{p-p_{l}} & \text{if }p_{l}<x\leq p\\ \frac{p_{r}-x}{p_{r}-p} & \text{if }p<x<p_{r} \end{array}\right..$$ (7)

where *p*_*l*_, *p*, and *p*_*r*_ denote the left, peak, and right endpoints of the triangle, respectively. The formulas for Fig 8 are as follows:

$$\mu_{\text{Far}}^{\text{UMF}}(x)=\left\{ \begin{array}{cc} 0 & \text{if }x<1.68\\ \frac{1.848-x}{1.848-1.68} & \text{if }1.68\leq x<1.848\\ 0 & \text{if }x\geq 1.848, \end{array}\right.$$ (8)

$$\mu_{\text{Far}}^{\text{LMF}}(x)=\left\{ \begin{array}{cc} 0 & \text{if }x<1.68\\ \frac{1.806-x}{1.806-1.68} & \text{if }1.68\leq x<1.806\\ 0 & \text{if }x\geq 1.806, \end{array}\right.$$ (9)

$$\mu_{\text{Moderate}}^{\text{UMF}}(x)=\left\{ \begin{array}{cc} 0 & \text{if }x<1.722\\ \frac{x-1.722}{1.89-1.722} & \text{if }1.722\leq x<1.89\\ \frac{2.058-x}{2.058-1.89} & \text{if }1.89\leq x<2.058\\ 0 & \text{if }x\geq 2.058, \end{array}\right.$$ (10)

$$\mu_{\text{Moderate}}^{\text{LMF}}(x)=\left\{ \begin{array}{cc} 0 & \text{if }x<1.764\\ \frac{x-1.764}{1.89-1.764} & \text{if }1.764\leq x<1.89\\ \frac{2.016-x}{2.016-1.89} & \text{if }1.89\leq x<2.016\\ 0 & \text{if }x\geq 2.016, \end{array}\right.$$ (11)

$$\mu_{\text{Close}}^{\text{UMF}}(x)=\left\{ \begin{array}{cc} 0 & \text{if }x<1.932\\ \frac{x-1.932}{2.1-1.932} & \text{if }1.932\leq x<2.1\\ 0 & \text{if }x\geq 2.1, \end{array}\right.$$ (12)

$$\mu_{\text{Close}}^{\text{LMF}}(x)=\left\{ \begin{array}{cc} 0 & \text{if }x<1.974\\ \frac{x-1.974}{2.1-1.974} & \text{if }1.974\leq x<2.1\\ 0 & \text{if }x\geq 2.1. \end{array}\right.$$ (13)

Fig 9 shows the membership functions for speed, with the input variable denoted as *y*. The input variable *y* is set within the range of 0 to 9, which allows for effective control of the vehicle’s speed based on the input variable *x*. This range is divided into six intervals and configured with values suitable for the 40m to 49m range. The formulas for these functions are presented as

$$\mu_{\text{Slow}}^{\text{UMF}}(y)=\left\{ \begin{array}{cc} 0 & \text{if }y<0\\ \frac{3.6-y}{3.6-0} & \text{if }0\leq y<3.6\\ 0 & \text{if }y\geq 3.6, \end{array}\right.$$ (14)

$$\mu_{\text{Slow}}^{\text{LMF}}(y)=\left\{ \begin{array}{cc} 0 & \text{if }y<0\\ \frac{2.7-y}{2.7-0} & \text{if }0\leq y<2.7\\ 0 & \text{if }y\geq 2.7, \end{array}\right.$$ (15)

$$\mu_{\text{Moderate}}^{\text{UMF}}(y)=\left\{ \begin{array}{cc} 0 & \text{if }y<0.9\\ \frac{y-0.9}{4.5-0.9} & \text{if }0.9\leq y<4.5\\ \frac{8.1-y}{8.1-4.5} & \text{if }4.5\leq y<8.1\\ 0 & \text{if }y\geq 8.1, \end{array}\right.$$ (16)

$$\mu_{\text{Moderate}}^{\text{LMF}}(y)=\left\{ \begin{array}{cc} 0 & \text{if }y<1.8\\ \frac{y-1.8}{4.5-1.8} & \text{if }1.8\leq y<4.5\\ \frac{7.2-y}{7.2-4.5} & \text{if }4.5\leq y<7.2\\ 0 & \text{if }y\geq 7.2, \end{array}\right.$$ (17)

$$\mu_{\text{Fast}}^{\text{UMF}}(y)=\left\{ \begin{array}{cc} 0 & \text{if }y<5.4\\ \frac{y-5.4}{9-5.4} & \text{if }5.4\leq y<9\\ 0 & \text{if }y\geq 9, \end{array}\right.$$ (18)

$$\mu_{\text{Close}}^{\text{LMF}}(y)=\left\{ \begin{array}{cc} 0 & \text{if }y<6.3\\ \frac{y-6.3}{9-6.3} & \text{if }6.3\leq y<9\\ 0 & \text{if }y\geq 9. \end{array}\right.$$ (19)

The fuzzy rules are formulated as shown in Table 3. N represents negative, Z stands for zero, and P denotes positive. The fuzzy rules and output range are fine-tuned through experimentation to ensure a consistent decrease in vehicle speed. The output range extends from 0 to 75.6, evenly divided into seven categories: NB, NM, NS, Z, PS, PM, and PB. When the vehicle speed is low, and the distance is large, a negative control torque is subtracted from the input torque to increase speed. Conversely, when the speed is high and the distance is short, a positive control torque is applied to decrease speed. The fuzzy rules are established based on these considerations.

**Table 3:** Fuzzy rules of interval type-2.

	Far	Moderate	Close
Slow	NM	NS	Z
Moderate	NM	Z	PS
Fast	Z	PS	PM

*A* and *B* represent the input variables for speed and distance, respectively. Each input variable is characterized by fuzzy sets *A*_*i*_, *B*_*j*_ and has membership functions $\mu_{Ai}$, $\mu_{Bj}$. The indices are represented as i,j=1,2,3.

$$\mu_{Ai}(x)\in A_{i}\equiv [\mu_{Ai}^{LMF}(x),\mu_{Ai}^{UMF}(x)],\;i=1,2,3,$$ (20)

$$\mu_{Bj}(y)\in B_{j}\equiv [\mu_{Bj}^{LMF}(y),\mu_{Bj}^{UMF}(y)],\;j=1,2,3.$$ (21)

UMF refers to the upper membership function, while LMF denotes the lower membership function.

In fuzzy inference, the degree of activation is expressed based on the rules as follows:

$$w_{k}^{UMF}=\mu_{Ai}^{UMF}(x)\cdot \mu_{Bj}^{UMF}(y),$$ (22)

$$w_{k}^{LMF}=\mu_{Ai}^{LMF}(x)\cdot \mu_{Bj}^{LMF}(y).$$ (23)

The degree of activation reflects the extent to which the inputs satisfy the rules. The product method is used for the AND operation, and all rule weights are set to 1. Here, *x* and *y* represent the values of the input variables, and *k* denotes the index of the rules.

The KM algorithm is used for type reduction. The output functions have a constant value *C* and are arranged in ascending order, as follows:

$$C_{\left(1\right)}\leq C_{\left(2\right)}\leq \ldots \leq C_{\left(n\right)}.$$ (24)

The left point *y*_*l*_ and the right point *y*_*r*_ indicate the potential range of output values. The initial values of *y*_*l*_ and *y*_*r*_ correspond to the minimum and maximum values of *C*, respectively. This is illustrated as follows:

$$y_{l}^{\left(0\right)}=\min_{k}C_{k},$$ (25)

$$y_{r}^{\left(0\right)}=\max_{k}C_{k}.$$ (26)

The KM algorithm iteratively converges these two values. The iterative calculation process is described as follows:

$$y_{l}^{\left(t+1\right)}=\frac{\sum_{k=1}^{L}C_{\left(k\right)}\;{\overset{―}{w}}_{\left(k\right)}+\sum_{k=L+1}^{n}C_{\left(k\right)}\;{\underset{―}{w}}_{\left(k\right)}}{\sum_{k=1}^{L}{\overset{―}{w}}_{\left(k\right)}+\sum_{k=L+1}^{n}{\underset{―}{w}}_{\left(k\right)},}$$ (27)

$$y_{r}^{\left(t+1\right)}=\frac{\sum_{k=1}^{R}C_{\left(k\right)}\;{\underset{―}{w}}_{\left(k\right)}+\sum_{k=R+1}^{n}C_{\left(k\right)}\;{\overset{―}{w}}_{\left(k\right)}}{\sum_{k=1}^{R}{\underset{―}{w}}_{\left(k\right)}+\sum_{k=R+1}^{n}{\overset{―}{w}}_{\left(k\right)}.}$$ (28)

*t* denotes the number of iterations. The switching points L and R are the largest indices of *C*_*k*_ that are less than or equal to $y_{l}^{\left(t\right)}$ and $y_{r}^{\left(t\right)}$ calculated in the current iteration. Therefore, they each satisfy the following:

$$C_{\left(L\right)}\leq y_{l}^{\left(t\right)}<C_{\left(L+1\right)},$$ (29)

$$C_{\left(R\right)}\leq y_{r}^{\left(t\right)}<C_{\left(R+1\right)}.$$ (30)

If $y_{l}^{\left(t+1\right)}$ sufficiently converges to $y_{l}^{\left(t\right)}$, then $y_{l}^{\left(t+1\right)}$ is set as the final left point *y*_*l*_. The same process is applied to *y*_*r*_.

After using the KM algorithm for type reduction, defuzzification is performed to achieve a crisp output. The calculation is performed as follows:

$$y_{\text{output}}=\frac{y_{l}+y_{r}}{2}.$$ (31)

The defuzzification output is used to control the torque to adjust the vehicle’s speed.

**Fig 10:**
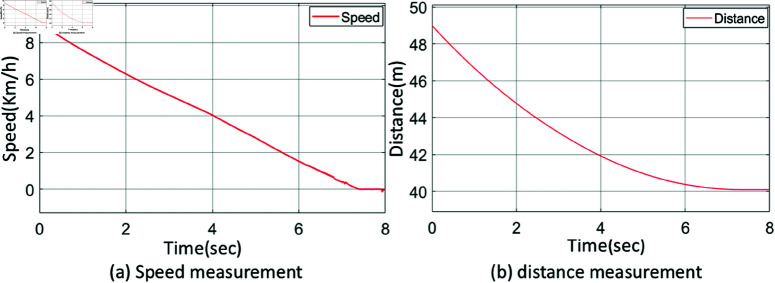
Output of the vehicle system: a) speed measurement, b) distance measurement.

A simulation based on experimentation is performed to move the vehicle from a distance of 49 m to a target located 40 m away. Here, 49 m is categorized as far, while 40 m is considered close. The vehicle starts at a speed of 9 km/h at 49 m and decelerates to 0 km/h upon reaching 40 m. Fig 10(a) shows the vehicle’s speed, while Fig 10(b) shows the distance between the vehicle and the specified target.

### Comparison of controllers for disturbance

**Fig 11:**
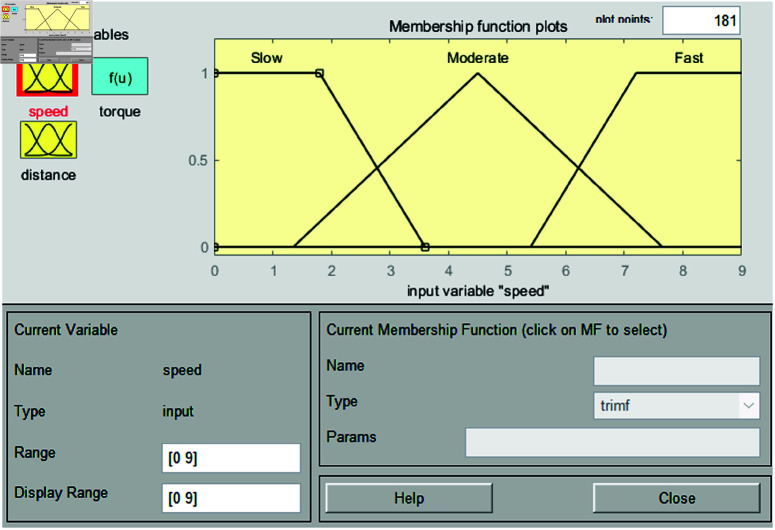
Type-1 membership function for speed.

**Fig 12:**
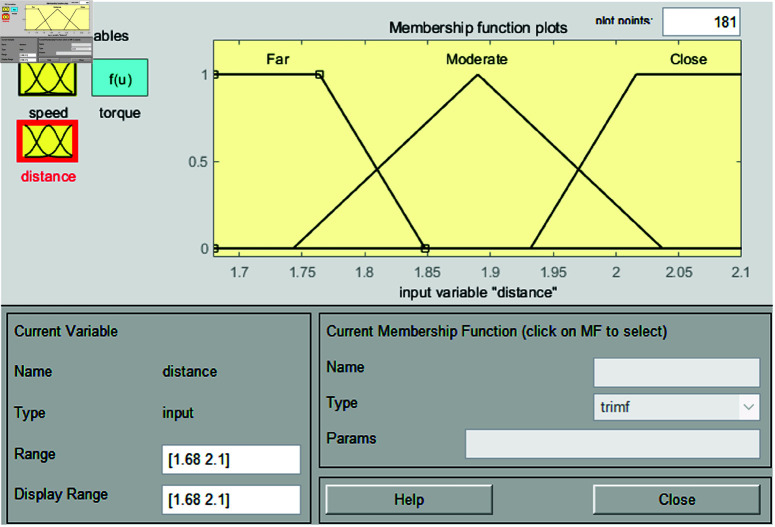
Type-1 membership function for distance.

To assess the performance of the interval type-2 fuzzy controller against disturbances, it is compared to a type-1 fuzzy controller. The rules for both controllers are set identically. The membership functions in the FIS main editor of MATLAB/SIMULINK are depicted in Figs 11 and 12. The FIS uses the Sugeno method for design. The AND operation in all rules uses the product method, while defuzzification is performed using the weighted average method. The output function ranges from 0 to 85.8 and is assigned to seven constant values: NB, NM, NS, Z, PS, PM, and PB. Additionally, the output function is applied according to the defined rules.

**Fig 13:**
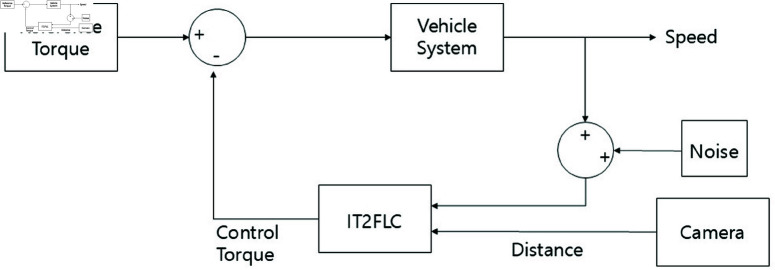
Vehicle system with disturbances.

The type-1 fuzzy controller and the interval type-2 fuzzy controller are integrated into the vehicle system. Two vehicle systems are evaluated with and without disturbances. Both systems begin at an initial speed of 9 km/h and decelerate to 0 km/h in 7 s. The disturbance is considered as noise from the sensor that measures the vehicle speed during this process. When passing the vehicle speed to the fuzzy controller as input, a random value following a normal distribution with a mean of 0 and a variance of 2 is added as noise. The model of the entire system, taking the disturbance into account, is shown in Fig 13.

**Fig 14:**
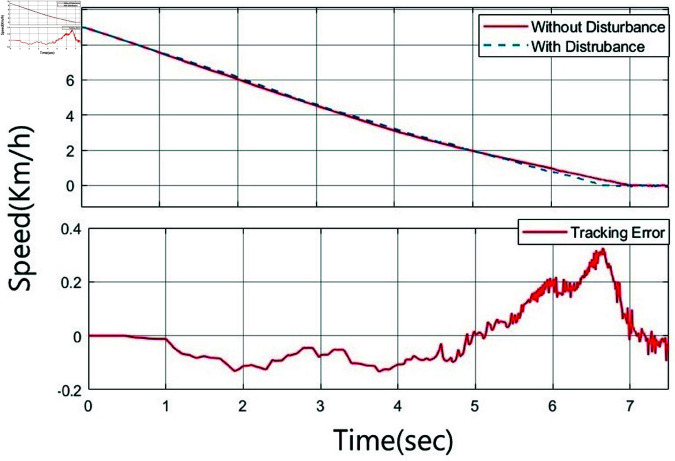
Vehicle speed and tracking error of the type-1 fuzzy controller in the presence of disturbances.

**Fig 15:**
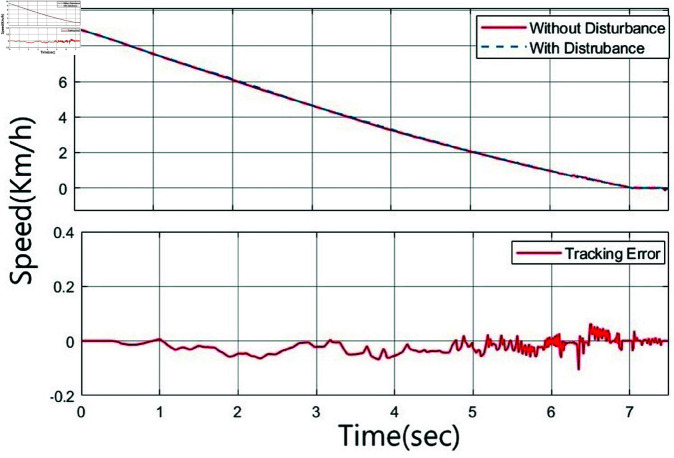
Vehicle speed and tracking error of the interval type-2 fuzzy controller in the presence of disturbances.

Figs 14 and 15 show the vehicle speed and disturbance tracking error for both the type-1 fuzzy controller and the interval type-2 fuzzy controller under both disturbed and undisturbed conditions. The disturbance tracking error is defined as the difference in speed between scenarios with disturbances and those without. It demonstrates that the tracking error of the type-1 controller is considerably higher than that of the interval type-2 controller. This finding highlights the superior performance of the interval type-2 fuzzy controller.

## Treatment of the limit of image data using an adaptive neuro-fuzzy inference system

Previous studies obtain vehicle distance data by capturing images using light. Therefore, acquiring a sufficient amount of vehicle distance data is a challenge. To address this issue, this study introduces an ANFIS. Using the artificial neural network in the ANFIS, existing distance data can be learned to develop a new input–output model. In this study, the ANFIS is trained using input–output data, where the vehicle speed and distance are used as the input to generate the control torque. This approach presents a method for designing an effective controller even when distance data are scarce.

Acquiring continuous distance data from headlamp reflection images is challenging. For instance, if image data are available at 1 m intervals from 41 to 49 m, it is impossible to determine the exact data for intermediate distances such as 41.5 m. To address this issue, the study uses ANFIS to develop a fuzzy controller capable of managing the vehicle even when data for specific distance ranges are missing. The ANFIS controller is developed using the neuro–fuzzy designer app in MATLAB/SIMULINK based on the Sugeno method.

The training data comprises input and output data from fuzzy controllers based on images collected between 41–43 m and 47–49 m. These data are used to create a fuzzy controller that covers the entire range from 41 to 49 m, even in the absence of data from 44 to 46 m.

**Fig 16:**
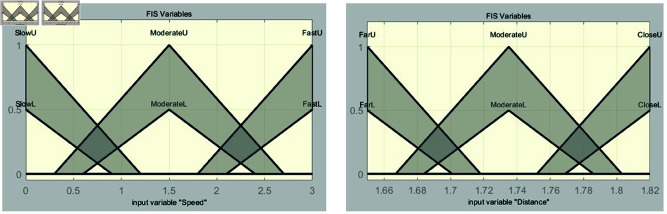
Membership functions for speed and distance in the 41–43 m section.

**Fig 17:**
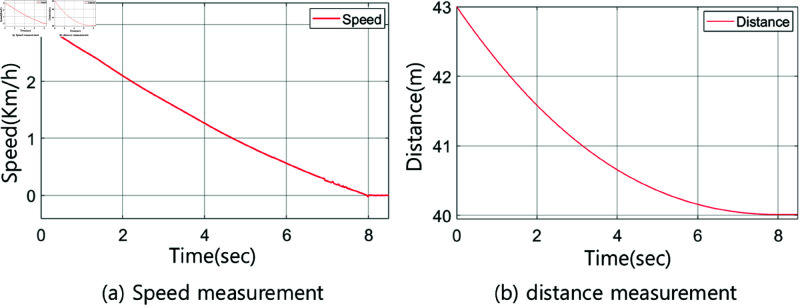
Output of the vehicle system for the 41–43 m section: a) speed measurement, b) distance measurement.

**Fig 18:**
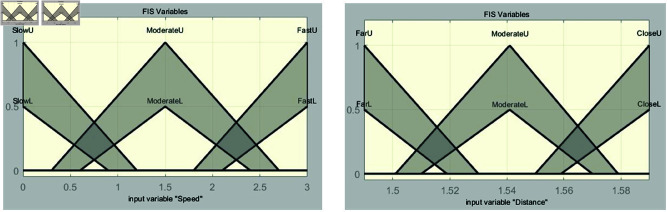
Membership functions for speed and distance in the 47–49 m section.

**Fig 19:**
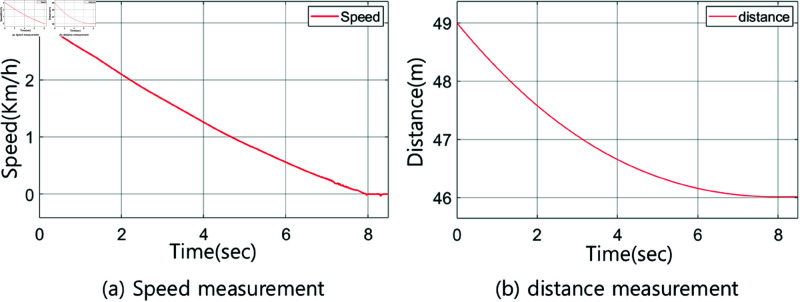
Output of the vehicle system for the 47–49 m section: a) speed measurement, b) distance measurement.

The rules for the fuzzy controllers in the ranges of 41–43 m and 47–49 m are identical to those presented in Table 3. The fuzzy controllers are designed using the interval type-2 method. Figs 16 and 17 show the membership functions of the fuzzy controller for the range of 41–43 m, along with graphs depicting the speed and distance of the vehicle systems. Meanwhile, Figs 18 and 19 provide detailed information about the fuzzy controller for the range of 47–49 m.

**Fig 20:**
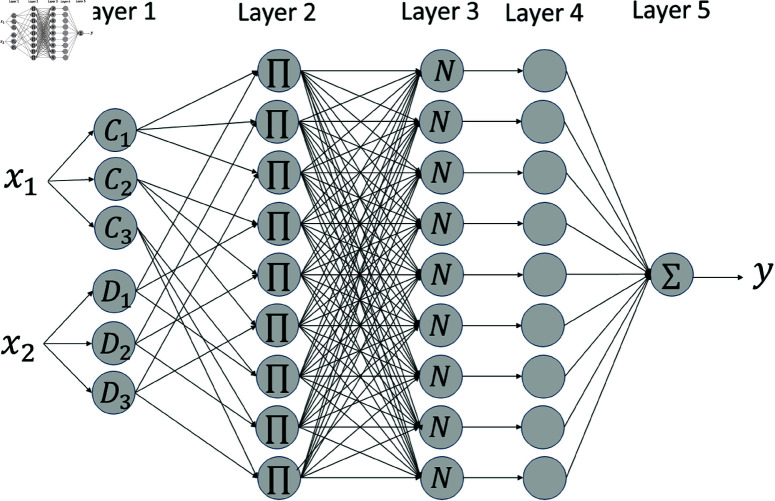
The five layers of the adaptive neuro–fuzzy inference system.

ANFIS is used to create a fuzzy controller by training it on data specific to each interval. Data are collected from the vehicle system at a sampling rate of 0.01 s over a duration of 8.5 s for the intervals of 41–43 m and 47–49 m. A total of 1,500 data points regarding distance, speed, and control torque are collected and used in the ANFIS training process. This training consists of 1,000 iterations and uses a hybrid optimization approach that combines the backpropagation algorithm with the least squares method. The design of ANFIS follows the Sugeno method, and its five-layer architecture is shown in Fig 20.

ANFIS is composed of five layers. The first layer serves as the fuzzification stage, where input variables are transformed into membership values of fuzzy sets. The input variables are *x*_1_ and *x*_2_, with their corresponding membership functions defined as *C*_*i*_ and *D*_*j*_. The values of these membership functions are shown in:

$$\mu_{C_{i}}(x_{1})=\left\{ \begin{array}{cc} 0 & \text{if }x_{1}<a_{i}\text{ or }x_{1}>c_{i}\\ \frac{x_{1}-a_{i}}{b_{i}-a_{i}} & \text{if }a_{i}\leq x_{1}<b_{i}\\ \frac{c_{i}-x_{1}}{c_{i}-b_{i}} & \text{if }b_{i}\leq x_{1}\leq c_{i} \end{array}\right.,\;i=1,2,3,$$ (32)

$$\mu_{D_{j}}(x_{2})=\left\{ \begin{array}{cc} 0 & \text{if }x_{2}<a_{j}\text{ or }x_{2}>c_{j}\\ \frac{x_{2}-a_{j}}{b_{j}-a_{j}} & \text{if }a_{j}\leq x_{2}<b_{j}\\ \frac{c_{j}-x_{2}}{c_{j}-b_{j}} & \text{if }b_{j}\leq x_{2}\leq c_{j} \end{array}\right.,\;j=1,2,3.$$ (33)

The parameters *a*, *b*, and *c* denote the start, midpoint, and endpoint of the triangular functions, respectively.

In the second layer, the activation levels for the rules related to the membership values of the input variables *x*_1_ and *x*_2_ are calculated. Each rule is represented by the product of the membership values of the input variables, as illustrated in:

$$w_{ij}=\mu_{C_{i}}(x_{1})\cdot \mu_{D_{j}}(x_{2}).$$ (34)

In the third layer, the normalized activation levels ${\bar{w}}_{ij}$ are determined by taking the ratio of each rule’s activation to the total activation levels of all rules. The activation levels for each rule are normalized as shown in:

$${\bar{w}}_{ij}=\frac{w_{ij}}{\sum_{k=1}^{3}\sum_{l=1}^{3}w_{kl}}.$$ (35)

In the fourth layer, the output for each rule of the Sugeno model is calculated. The membership functions for the output variables are defined as constants. *z*_*ij*_ represents the output membership function for the rule related to the membership functions *C*_*i*_ and *D*_*j*_ of the input variables *x*_1_ and *x*_2_. The rule’s output is determined by multiplying the rule’s activation levels by the value of its corresponding output function. Specifically, *y*_*ij*_ indicates the output value for this rule. The rule’s output is calculated as illustrated in:

$$y_{ij}={\bar{w}}_{ij}\cdot z_{ij}.$$ (36)

The fifth layer performs the defuzzification process to determine the overall output value. A weighted average method is used for this defuzzification. The computation process is presented in:

$$y=\sum_{i=1}^{3}\sum_{j=1}^{3}{\bar{w}}_{ij}\cdot z_{ij}.$$ (37)

In this study, an ANFIS is used to address the issue of missing data at specific intervals. The learning algorithm is described as follows. In the fuzzification stage, fuzzy membership functions are applied to the input values. Since this study is based on the Sugeno model, the output is expressed as a linear function. In the second layer, the activation degree of each fuzzy rule is calculated, and the weight of each rule corresponding to the distance and speed is determined. In the third layer, the weight of each rule is normalized by dividing it by the sum of all rule weights, which ensures balanced rule representation. In the fourth layer, parameters are used to compute the output. Finally, in the fifth layer, the final control torque is generated.

**Fig 21:**
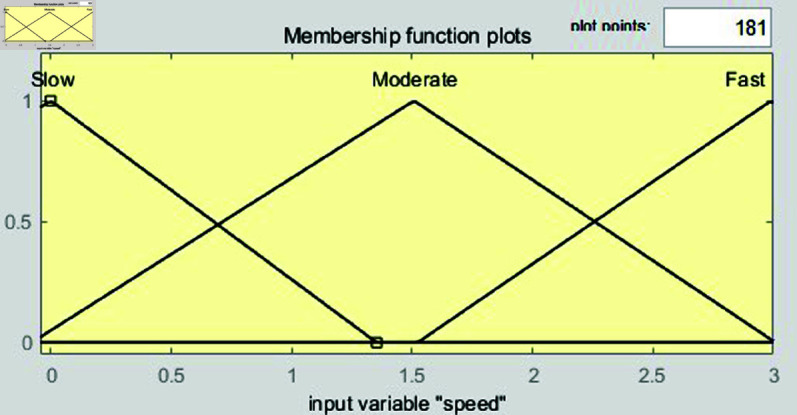
Membership functions for speed generated by the adaptive neuro–fuzzy inference system.

**Fig 22:**
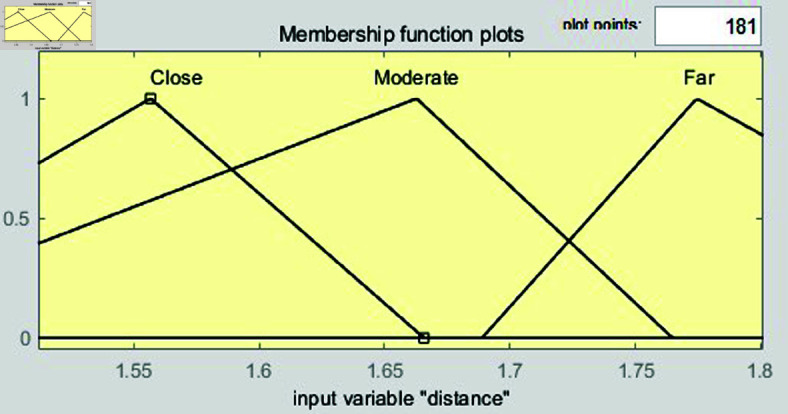
Membership functions for distance generated by the adaptive neuro–fuzzy inference system.

**Fig 23:**
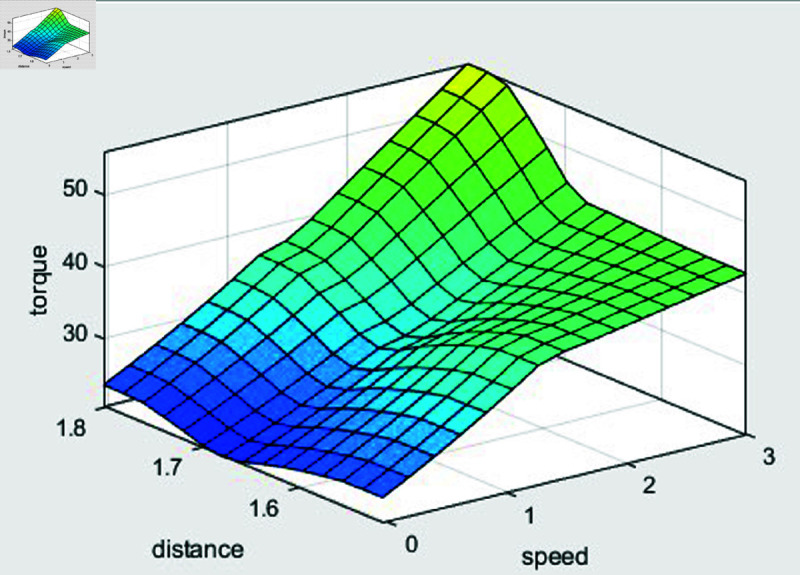
Input and output areas of the adaptive neuro–fuzzy inference system controller.

ANFIS processes its five layers for a specific number of iterations to adjust weights and rule parameters according to the inputs and outputs. The ANFIS combines fuzzy systems and neural networks, providing high accuracy even in complex input-output relationships. In addition, by learning from training data, the ANFIS generates rules and improves real-time computational capability in various environments. The fuzzy rules are presented in Table 4. The membership functions for speed and distance are shown in Figs 21 and 22, respectively. Fig 23 shows the input–output regions.

**Table 4:** Fuzzy rules generated by the adaptive neuro–fuzzy inference system.

	Far	Moderate	Close
Slow	NB	NM	NS
Moderate	NZ	Z	PZ
Fast	PS	PM	PB

**Fig 24:**
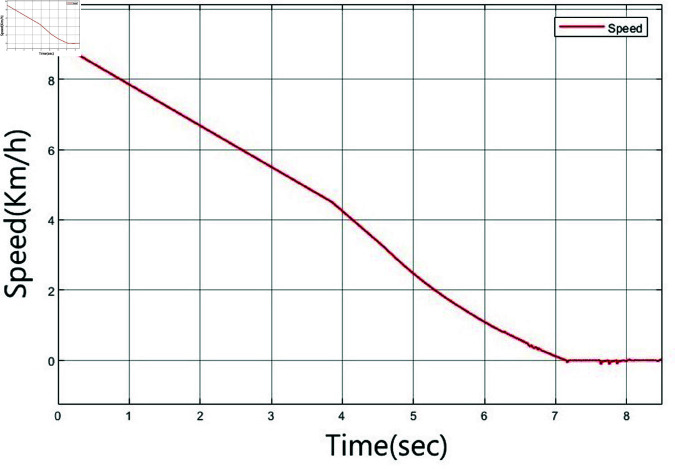
Output speed of the vehicle system with an ANFIS controller.

**Fig 25:**
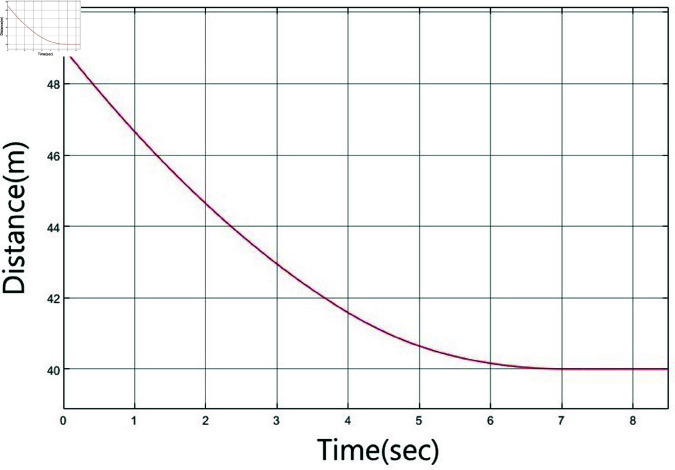
Distance variation of the vehicle system with an ANFIS controller.

The designed controller has been integrated into the vehicle system, as illustrated in Fig 6. Simulations based on experiments are performed in the 40–49 m range. Fig 24 shows the vehicle’s speed, while Fig 25 shows the distance of the vehicle to a specific target. Despite a lack of sufficient image data for distance, the speed of the vehicle successfully decreases, and it is confirmed that the vehicle stops at the target point of 40 m.

## Discussion

This study investigated two advanced control strategies an interval type-2 fuzzy controller and an ANFIS-based controller to address key challenges in autonomous driving systems. The interval type-2 fuzzy controller effectively mitigated noise from vehicle speed sensors by incorporating a more robust representation of fuzzy membership values, while the ANFIS-based controller provided a solution for handling insufficient distance data by leveraging existing data.

This study also emphasized the potential of simplifying autonomous driving systems by using cameras alone for distance estimation, rather than relying on complex sensor combinations like lidar and radar. While this approach reduces costs and processing demands, its feasibility has not been validated in diverse environments or real-world road conditions.

Some limitations were identified during experimentation. For instance, the ANFIS controller showed inconsistencies in reducing vehicle speed during early ranges, indicating the need for algorithmic improvements. Additionally, only speed sensor noise was modeled as a disturbance, leaving other potential disturbances unexplored. These issues suggest areas for refinement and future development to enhance the controllers’ overall effectiveness and reliability.

## Conclusion

This study successfully designed and implemented two controllers to improve autonomous driving performance. The interval type-2 fuzzy controller demonstrated strong robustness against sensor noise, while the ANFIS-based controller effectively addressed the challenge of insufficient distance data. Both controllers achieved the objective of stopping the vehicle at its destination, proving their practicality for vehicle control applications.

Future work will focus on validating these methods in a variety of environments and under real-world road conditions. This includes conducting experiments in different locations, improving light detection algorithms for image-based distance estimation, and performing real-time vehicle control tests with dynamic headlamp reflection processing. Addressing limitations in the ANFIS controller’s speed control performance and expanding the range of modeled disturbances will further enhance the system’s reliability and applicability. In addition, an explicit model predictive control approach will be used to design a controller with high performance in real-time computation. The performance of this controller will be compared with that of existing controllers, and strategies will be explored to integrate the strengths of each controller into the vehicle system.
